# Decadal advances and future prospects in subunit vaccine development against *Streptococcus suis* infection

**DOI:** 10.3389/fimmu.2025.1680732

**Published:** 2025-09-19

**Authors:** Lihua Fang, Jie Ning

**Affiliations:** ^1^ Department of Endocrinology, Shenzhen Longhua District Central Hospital, Shenzhen, Guangdong, China; ^2^ Department of Medical Laboratory, Shenzhen Longhua District Central Hospital, Shenzhen, Guangdong, China

**Keywords:** *Streptococcus suis*, subunit vaccines, adjuvants, cross-protection, vaccine delivery

## Abstract

*Streptococcus suis* (SS), particularly serotype 2 (SS2), is a significant zoonotic pathogen causing severe disease in swine and humans. High genetic diversity and antibiotic resistance complicate vaccine development. We firstly synthesize the pivotal advances in SS subunit vaccine design over the past ten years, thereby establishing a foundation for guiding future rational vaccine development. Promising candidates, including pS-Lpp-SaoA (delivered via OMVs), SaoA (via live vectors), IgA1 protease, rIde-10, rIde-14009-1, Enolase, 6-GPD, 38-BP-Enol, and multi-antigen formulations (MRP/GAPDH/DLD or SLY/Enolase/Sbp), elicit robust immune responses (high IgG/IgA titers) and confer up to 100% protection against lethal SS2 challenge in murine and porcine models. Cross-protection against heterologous serotypes (e.g., SaoA and Enolase delivered via *S. Choleraesuis*) is observed. Future efforts should prioritize: discovery of conserved antigens, optimization of delivery platforms/adjuvants, and translational validation in pigs to achieve broad, durable immunity.

## 1 Introduction

**Figure 1:**
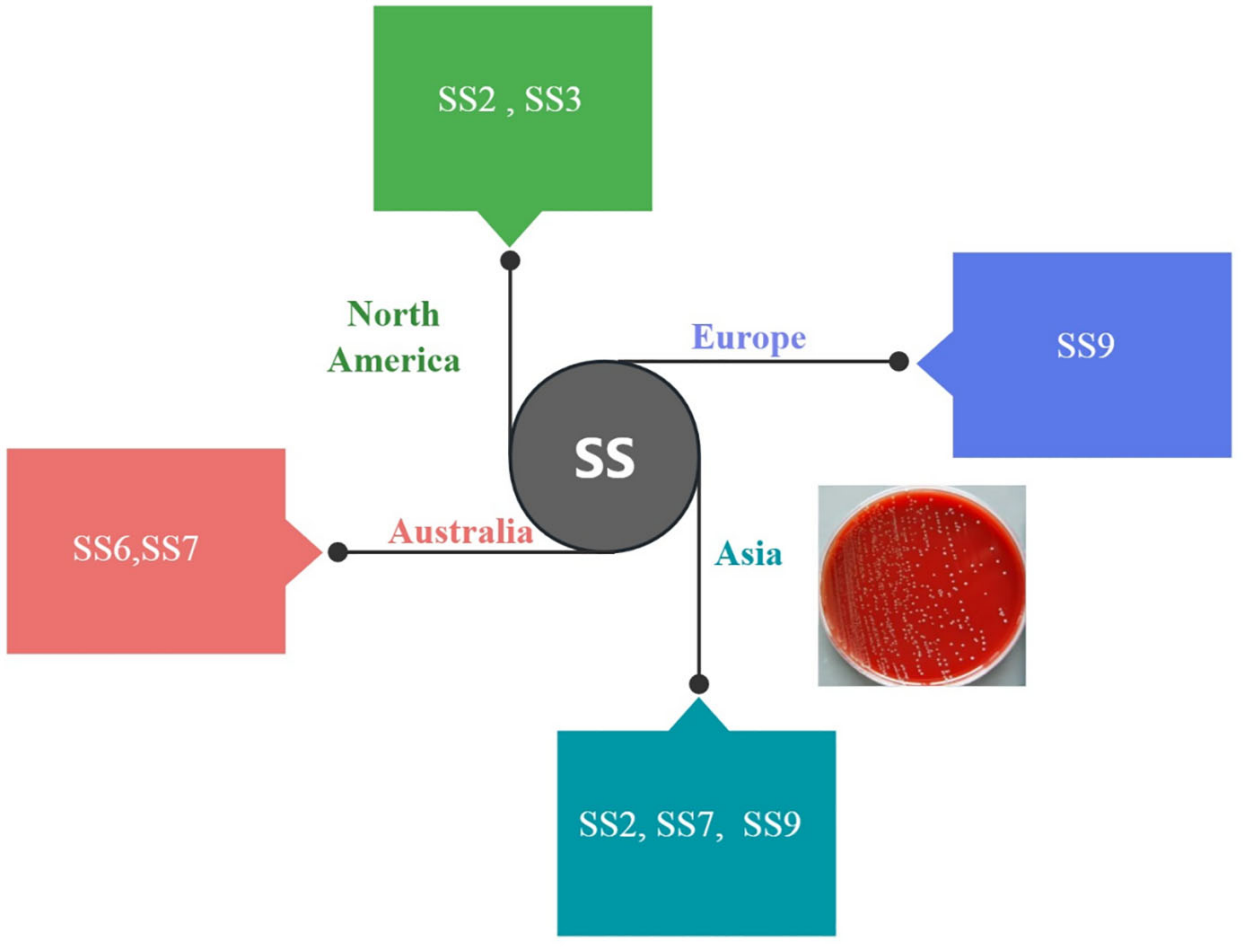
Global distribution of predominant *Streptococcus suis* serotypes in human infections. Pie charts illustrate the relative frequency of SS serotypes most commonly associated with human cases across major geographic regions. The notable regional variation in serotypes underscores the challenge in developing a universally effective vaccine.

*Streptococcus suis* especially serotype 2 (SS2) is a globally distributed zoonotic pathogen causing significant economic losses in the swine industry and posing a threat to human health (1). Since its initial report in the Netherlands in 1951 (2), SS2 has been identified in humans, particularly in those who consumed contaminated pork in some Asian countries, leading to severe conditions such as sepsis, pneumonia, meningitis, and toxic shock (3, 4). Notably, outbreaks in China in 1998 and 2005 resulted in numerous fatalities (5). It is also a highly diverse pathogen with multiple serotypes circulating globally, making vaccine development challenging. In North America, SS2 and SS3 are dominant, while SS9 is more common in Europe, SS6,SS7 predominate in Australia (6) and SS2, SS7, and SS9 are frequently reported in human cases in Asia (7), as shown in **Figure 1**. This significant regional serotype diversity poses a major challenge for developing vaccines capable of providing broad cross-protection (8). Traditional inactivated vaccines offer limited cross-protection, with protection rates around 70% against homologous strains and lower against heterologous strains (9).

SS2 exhibits varying antibiotic resistance patterns across regions, complicating control efforts. In Europe, resistance to lincosamides, macrolides, and tetracyclines is common, while β-lactams remain effective (10, 11). In Asia, resistance to these antibiotics is high and increasing, with emerging resistance to sulfonamides, aminoglycosides, and fluoroquinolones (12). North America shows similar resistance profiles to Europe but with lower resistance to β-lactams and amphenicols (13). From 2005 to 2021, sporadic human cases were consistently reported in Shenzhen, China, with strains showing resistance to multiple antibiotics (14). The high pathogenicity and multidrug resistance of SS2 pose a severe challenge to public health, highlighting the need for effective vaccines.

It employs a variety of virulence factors to infect pigs and humans (15–17), including adhesins (e.g., MRP, EF, FBPS, Lmb) (18), the toxin suilysin (SLY) (19), and immune evasion factors (e.g., IgdE, IdeSsuis, HP1022) (20). These virulence markers may contribute to its complex pathogenesis and ability to resist host defenses (21, 22). By 2015, despite extensive research, traditional SS vaccines (notably inconsistent bacterins and safety-challenged live-attenuated candidates) had largely failed to deliver reliable, cross-protective solutions against this economically devastating swine pathogen and emerging zoonotic threat (7). While subunit vaccines emerged as a promising alternative due to their safety profile and identification of numerous candidate antigens (21–23), critical barriers remained unresolved: efficacy was often limited to homologous strains, heavily dependent on specific adjuvants to induce essential opsonizing antibodies, and crucially lacked proven broad-spectrum protection across the pathogen’s highly diverse serotypes and sequence types.

Addressing these gaps, this review synthesizes advances from the past decade in SS subunit vaccine development, focusing on antigen discovery, rational design for immune targeting, the optimization of delivery platforms (e.g., viral vectors, bacterial vectors, OMVs) (24) and adjuvant strategies. It serves as a foundation for accelerating novel vaccines that overcome historical limitations.

## 2 Single subunit vaccines

Subunit vaccines have become a key strategy against SS, as they can induce strong and specific immune responses against this pathogen. The core of these vaccines lies in utilizing highly immunogenic antigens derived from SS, produced through the single expression in systems such as *E. coli* or other engineered bacterial strains or viral expression systems. Nevertheless, it is critical to emphasize that these antigens must not only be highly immunogenic but also highly conserved across different SS strains to ensure broad protection.

### 2.1 Sao antigen

The Sao protein (Surface-anchored protein), a highly conserved antigen (>94.2% homology across major *S. suis* serotypes), has been a focal point in SS vaccine research (**Table 1**). Its immunogenicity, conversely, is critically dependent on the delivery platform and adjuvant formulation. Initial evaluation of recombinant Sao-L (rSao-L) produced in bioreactors established its immunogenicity, inducing robust antigen-specific antibodies and cellular responses (increased CD8+ and CD4+/CD8+ DP T cells) in pigs, conferring a 60% reduction in lesions upon heterologous challenge; otherwise, this level of protection, although statistically significant, indicated substantial room for improvement. Furthermore, the reliance on a water-in-oil-in-water (w/o/w) emulsion adjuvant raised potential safety concerns regarding granuloma formation at the injection site (25).

**Table 1:** Summary of adjuvants’ advantages and disadvantages.

Adjuvant name	Advantages	Disadvantages
Freund’s Complete/Incomplete Adjuvants	Strong enhancement of immune responses; high IgG levels	Potential for local inflammatory reactions; generally not suitable for human use
Quil-A	Enhances mucosal and systemic immune responses	Potential for local reactions; dose optimization needed
TiterMax Gold	Significant enhancement of IgG responses, especially for CPS	High cost; may require cold chain storage
Stimune	Enhances IgG responses; provides good protection	Dependent on antigen type; may need combination with other adjuvants
ISA 206 VG	Suitable for multi-epitope vaccines; provides good protection	Potential for local reactions; formulation optimization needed
Aluminum Hydroxide (AlOH)	Low cost; widely used	Limited immune enhancement; higher doses may be needed
Montanide ISA206	Low cost; widely used	Limited immune enhancement; formulation optimization needed
Polygen™	Low cost; widely used	Limited immune enhancement; formulation optimization needed

In a subsequent refinement, a maternal vaccination strategy was employed: sows were immunized with a combination of recombinant Sao protein (rSao) and an inactivated bacterin. This approach significantly improved protection in piglets, demonstrating a 75-81% reduction in lesions upon challenge. This protection was mediated by the transfer of passive immunity from vaccinated sows to their offspring via colostrum. However, a limitation was observed, as the antibody titers in piglets waned by 6 weeks post-partum (26). A technologically advanced approach using engineered OMVs displaying lipidated SaoA (Lpp-SaoA fusion) leveraged intrinsic adjuvanticity, inducing superior, balanced immune responses and exceptional protection (up to 100% survival) in mice without external adjuvant, although porcine evaluation is pending (27). Live-attenuated *Salmonella* vectors (e.g., rSC0016, rSC0012) effectively delivered SaoA, inducing robust mucosal and systemic immunity and conferring strong homologous and heterologous (SS7) protection in pigs, with rSC0012 showing an improved safety profile (28, 29). The rSC0016 vector has been successfully utilized to deliver a variety of heterologous antigens, such as SaoA and Enolase from *S. suis* (28) and P42/P97 from *Mycoplasma hyopneumoniae*, inducing protective immune responses in both murine and porcine models (30, 31). This underscores its potential as a versatile and effective vaccine platform for swine respiratory diseases. Paradoxically, despite inducing cross-reactive opsonophagocytic antibodies (OPA) against multiple serotypes (SS2, SS7, SS9, SS1/2) via the *Salmonella* vector, monovalent SaoA provided limited protection (e.g., only 20% survival against SS9 in mice). This disconnect between OPA titers and *in vivo* efficacy highlights the antigen’s formulation-dependent variability and suggests potential issues with antibody functionality or undiscovered serotype-specific epitope variations (32). Thus, while Sao protein shows promise as a conserved target, achieving consistent, broad cross-protection and optimal safety remains challenging, contingent on advanced delivery platforms, including significant challenges in biosafety, manufacturing scalability, and regulatory acceptance, which must be addressed before they can replace traditional bacterins in the field.

### 2.2 Capsular polysaccharide antigen

Capsular polysaccharide (CPS) from *S. suis* is a key virulence factor, but its poor immunogenicity has posed significant challenges for vaccine development. Calzas et al. (33) found that CPS-specific antibody responses in infected animals are often absent or only slightly elevated compared to non-infected animals, primarily consisting of low-titer IgM. In contrast, experimental infections using live virulent SS2 strains (such as the European P1/7 or North American 89-1591) consistently induced robust immune responses in both mice and pigs. These responses were characterized by strong, isotype-switched IgG antibodies targeting bacterial proteins and exhibited memory features upon re-challenge. While the antibody response directed against the CPS was markedly impaired. This anti-CPS response was weak or often undetectable, predominantly consisting of low-titer IgM. Critically, it showed no significant isotype switching to IgG in either animal model and elicited only a minimal memory boost upon re-exposure (33). This CPS-specific unresponsiveness was independent of the infecting strain’s geographic origin or TLR2 signaling, underscoring the inherent poor immunogenicity of *S. suis* CPS *in vivo* and directly explaining the limitations of traditional bacterins.

To address these challenges, researchers have explored CPS glycoconjugate vaccines. Goyette-Desjardins et al. demonstrated the successful development and protective efficacy of a SS2 capsular polysaccharide glycoconjugate vaccine. By coupling purified, depolymerized, and oxidized CPS to tetanus toxoid (TT) and formulating it with emulsifying adjuvants (Stimune or TiterMax Gold), they induced robust, T cell-dependent immune responses in mice and pigs. This included high levels of IgM and isotype-switched IgG antibodies (including IgG1, IgG2b, IgG2c, IgG3 in mice and IgG1 in pigs) specific to the CPS, which exhibited functional *in vitro* opsonophagocytic activity (64-98% bacterial killing in mice). Crucially, the conjugate vaccine provided significant protection (70% survival) against a lethal intraperitoneal challenge with virulent SS2 in pigs, comparable to a commercial bacterin vaccine (72% survival), and significantly reduced clinical signs (abnormal behavior, lameness) and bacterial recovery from joints (34). Additionally, serotype 3 CPS was shown to induce robust opsonizing IgG responses in mice when adjuvanted with TiterMax Gold, while CPS from other serotypes (7, 8, and 9) failed to elicit significant antibody responses (21).

### 2.3 IgA1 protease and IgM protease antigen

IgA1 protease and IgM protease have emerged as promising candidates for *S. suis* vaccines due to their roles in bacterial pathogenesis and immune evasion. Recombinant IgA1 protease (rIgAP) from SS2 has been shown to induce high levels of IgG antibodies and provide complete protection against lethal SS2 challenge in mice when combined with a Marcol 52-based adjuvant (35). This finding confirms that IgA1 protease is expressed on the bacterial surface, making it a potential surface protective antigen. Thus, its efficacy in pigs remains unconfirmed, highlighting the need for further research to evaluate its potential in relevant animal models (36). Similarly, the IgM protease Ide-*S.suis*, which disrupts the classical complement pathway by cleaving IgM, has been shown to provide protection against a highly virulent serotype 9 strain in pigs (37) showed that Ide-*S.suis*, an IgM protease, disrupts the classical complement pathway by cleaving IgM, aiding bacterial evasion in pigs. Vaccination with the recombinant IgM-degrading enzyme (rIde) induced specific IgG antibodies and reduced bacterial survival in blood, leading to 100% survival in vaccinated piglets challenged with a lethal dose of *S. suis* cps9 strain (38). But vaccinated pigs exhibited signs of morbidity, such as fever and lameness, indicating partial protection.

Expanding on these insights, the IgM protease vaccine based on the Ide-*S.suis* gene has been shown to induce serotype-independent protection in pigs against multiple *S. suis* strains expressing group A IgM protease (39). This vaccine was effective in both piglet and gilt vaccination studies, providing protection against various strains, including st1, st2, st9, and st14. No protection was observed against strains expressing group B IgM protease, highlighting the need for a more comprehensive antigenic coverage. The study also developed a qPCR test to classify *S. suis* strains based on their IgM protease groups, which could aid in predicting vaccine efficacy. Additionally, research by Dolbec et al. (40) has further explored the potential of IgM-targeting strategies in vaccine development, emphasizing the necessity of enhancing IgM-focused approaches to improve host defense against *S. suis*. While IgA, IgG, and IgM proteases have shown promise as vaccine candidates, several limitations remain, including serotype-specific efficacy and incomplete protection against certain strains. Further *in vivo* studies in relevant animal models are needed to improve cross-protection and confirm overall efficacy.

### 2.4 ABC transporter antigens

PstB was identified as a highly conserved protein with nearly 100% amino acid sequence identity across various *S. suis* isolates. Immunization with recombinant PstB (rPstB) induced high levels of IFN-γ and IL-4, indicating strong Th1 and Th2 immune responses. Mice immunized with rPstB showed significant protection against challenges with SS2 (87.5% survival), SS7 (62.5% survival), and SS9 (87.5% survival). A multi-epitope construct of PstB provided poor protection (12.5% survival) against all tested serotypes (41).

S-ABC was found to be highly conserved across multiple SS strains, with 97% amino acid sequence identity. Mice immunized with recombinant S-ABC (rS-ABC) exhibited strong antigen-specific antibody responses and significant production of IFN-γ and IL-4, indicating robust Th1 and Th2 immune responses. The vaccine conferred high levels of protection against challenges with SS2 (87.5% survival) and 9 (100% survival), and moderate protection against serotype 7 (50% survival). In contrast, a multi-epitope construct of S-ABC provided lower protection (25%-37.5%). These results demonstrate that full-length rS-ABC is a promising candidate for a universal subunit vaccine against multiple SS serotypes, though further research is needed to optimize its efficacy and explore its potential in broader serotype coverage (42).

Moreover, Zhang et al. (2021) (43) synthesized oligosaccharides resembling the CPS of *S. suis* serotypes 2, 3, 9, and 14, identifying lead antigens with the potential to elicit immune responses. While this work laid the groundwork for glycoconjugate vaccine development, it remains in the early stages, with no *in vivo* efficacy data yet available. Similarly, Singh et al. (2022) (44) reported the synthesis of oligosaccharides for *S. suis* serotype 18, overcoming challenges in synthesizing the penta-saccharide repeating unit. This research provides a foundation for future glycoconjugate vaccine development but has not yet evaluated the immunogenicity or protective efficacy of the synthesized antigens *in vivo*.

### 2.5 The enolase antigen

Recombinant *S. suis* Enolase (SsEno) and Dipeptidyl Peptidase IV (DPPIV) subunit vaccines, although expressed by 86% and 88% of field strains respectively and inducing strong antibody responses in mice, failed to provide significant protection against a lethal serotype 2 challenge in an outbred (CD-1) mouse model (45). This lack of protection was consistent regardless of the adjuvant used (Quil-A^®^, Polygen™, Stimune^®^, or Montanide™ ISA 50 V2), demonstrating that adjuvant optimization alone could not confer efficacy to these antigens under these experimental conditions. However, a study by Li et al. using a recombinant *S. Choleraesuis* vector (rSC0016) carrying the enolase antigen achieved 100% protection against SS2 and SS9, and 80% protection against SS7 in mice. This study evaluated a live attenuated *Salmonella enterica serovar Choleraesuis* vector (rSC0016) delivering a conserved surface protein enolase as a potential universal vaccine against multiple serotypes of *S. suis*. The enolase, a highly conserved surface protein present in all *S. suis* serotypes, was expressed by the rSC0016(pS-Enolase) vaccine strain. The results showed that the vaccine strain effectively colonized the lymphatic tissues of mice and elicited strong mucosal, humoral, and cellular immune responses against enolase (46).

This study evaluated a live attenuated *Salmonella enterica serovar Choleraesuis* vector (rSC0016) delivering a conserved surface protein enolase as a potential universal vaccine against multiple serotypes of SS. The enolase, a highly conserved surface protein present in all SS serotypes, was expressed by the rSC0016(pS-Enolase) vaccine strain. The results showed that the vaccine strain effectively colonized the lymphatic tissues of mice and elicited strong mucosal, humoral, and cellular immune responses against enolase. These findings suggest that the rSC0016(pS-Enolase) vaccine is a promising candidate for a universal vaccine against multiple SS serotypes, offering a balance between host safety and immunogenicity.

Drawing from these observations, Li et al. (32) constructed a dual-antigen expression cassette using *S. Choleraesuis*, which provided broad protection against multiple *S. suis* serotypes (2, 7, 9, and 1/2) in mice, with protection rates ranging from 80% to 100%. This dual-antigen approach, combining Sao and Enolase, demonstrated the potential to enhance protective immunity by leveraging the strengths of multiple conserved antigens. The results highlight the importance of combining antigens to develop a universal vaccine against multiple *S. suis* serotypes, suggesting that a multi-antigen strategy may be more effective in providing broad-spectrum protection.

### 2.6 Other subunit antigens

Other subunit vaccines, such as 6-PGD, PDH, SsnA, EF-Tu, PrsA, and SBP2, have shown significant immunogenicity and varying degrees of protection in mice and rabbits.

Researchers developed a live attenuated *Salmonella enterica serovar Choleraesuis* vaccine vector (rSC0011), incorporating regulated delayed attenuation and regulated delayed antigen synthesis, to deliver the 6-phosphogluconate dehydrogenase (6-PGD) protein from SS2. The vaccine strain rSC0011 exhibited significant attenuation and enhanced colonization in mice lymphoid tissues compared to the licensed vaccine strain C500. Mice immunized with rSC0011(pS6-PGD) developed strong immune responses, including high levels of serum IgG and mucosal IgA antibodies against 6-PGD and *Salmonella* antigens. The vaccine conferred 90% protection against intraperitoneal challenge with a lethal dose of SS2, demonstrating its potential as an effective vaccine candidate (47).

Pyruvate dehydrogenase (PDH), a biofilm-associated protein of SS, is also a subunit vaccine candidate. The recombinant PDH (rPDH) was expressed and purified from *E. coli* and used to immunize mice with the ISA206 adjuvant. Results showed that PDH had high sequence conservation among different *S. suis* serotypes and strong immunogenicity, inducing high levels of specific antibodies (up to 1:409,600 titer) and significant expression of immune-related genes (CD4, CD8, IFN-γ, and IL-6) in mice spleens. Mice immunized with rPDH or inactivated bacteria exhibited 70% and 60% survival rates, respectively, against a lethal dose of *S. suis* ZY05719, with reduced pathological damage in vital organs like liver, brain, and spleen. Additionally, PDH antiserum significantly inhibited *S. suis* growth and biofilm formation *in vitro*. Conversely, the study was limited to a single serotype 2, and its efficacy across other serotypes remains to be determined (48). Additionally, vaccines based on other proteins like SsnA (49), EF-Tu (50), PrsA (51), and SBP2 (52) have shown varying degrees of protection in mice and rabbits. In especial, the rSsnA + ALOH vaccine achieves a protection rate of 91.25% against SS in mice, significantly reducing mortality and bacterial counts. Some proteins, like PrsA and SBP2, have shown partial cross-protection against multiple serotypes. Nonetheless, most studies are limited to murine models, and their efficacy in natural hosts (e.g., pigs) remains untested.

In the viral vector domain, the BoHV-4/GMD vaccine achieved a protection rate of 71.4% against SS2 challenge, while BoHV-4/SLY provided only 12.5% protection. The study demonstrated that BoHV-4/GMD induced higher levels of antibody-mediated phagocytic activity against SS2, SS7, and SS9 compared to BoHV-4/SLY. Although the study provides promising results for BoHV-4/GMD as a potential vaccine candidate, the findings are limited by the use of a rabbit model, which may not fully replicate the immune response in pigs. Additionally, the study did not directly evaluate the protective efficacy against SS7 and SS9 due to the lack of a suitable rabbit model for these serotypes (53).

## 3 Multi-subunit vaccines

**Figure 2:**
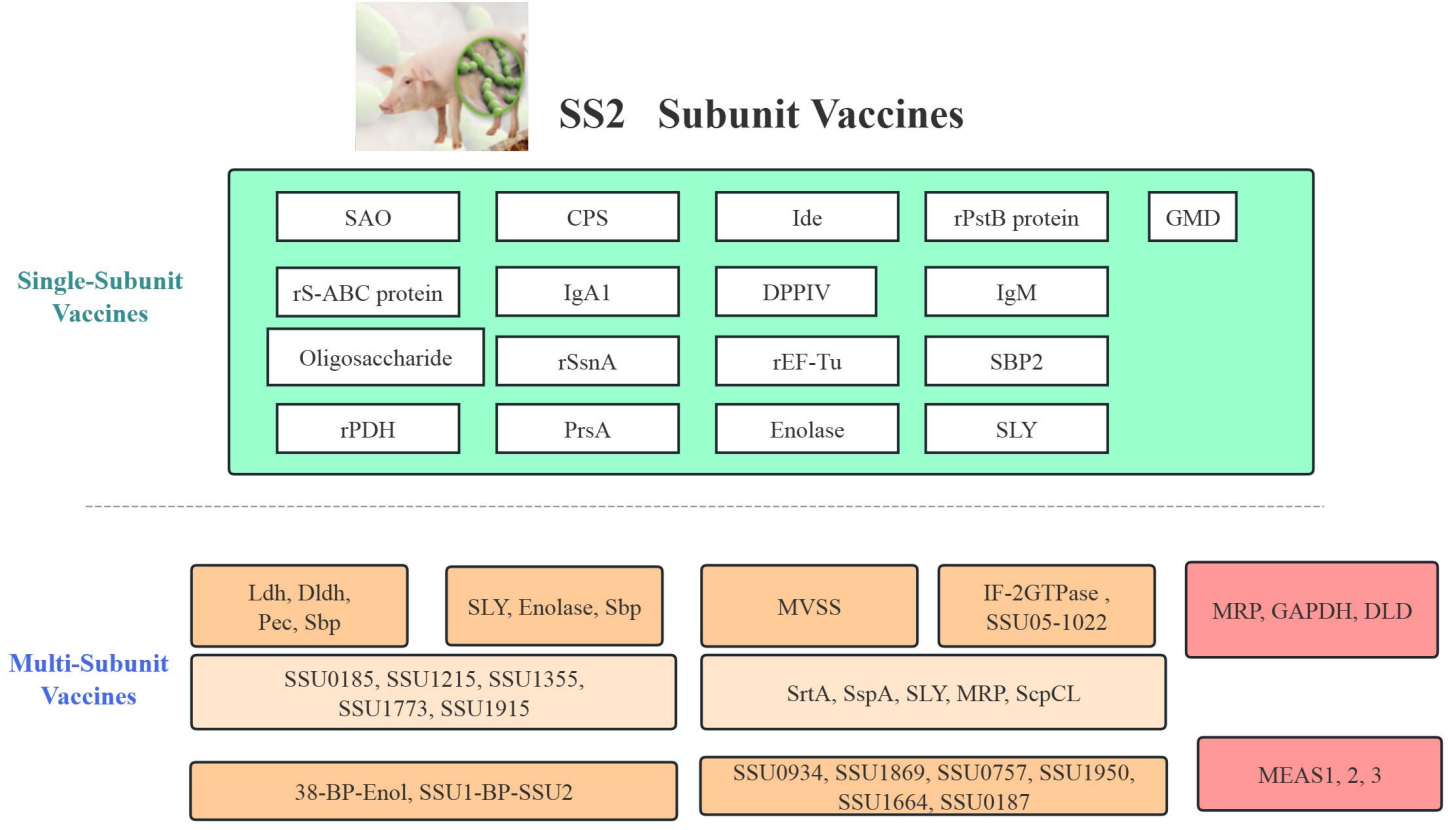
Comprehensive classification and evaluation of *Streptococcus suis* subunit vaccines and associated animal models. This figure summarizes various vaccine strategies including single antigen vaccines and multi-subunit formulations.

Multi-subunit vaccines have shown promise in providing broader protection against *S. suis* by combining multiple antigens to enhance immune responses and increase cross-protection across different serotypes (**Figure 2**). Initial efforts focused on membrane-associated proteins such as Ldh, Dldh, Pec, and Sbp, with Sbp showing the most promising results, eliciting strong humoral immune responses and providing protection against lethal challenges in mice (54). Subsequent studies identified additional proteins (SSU0185, SSU1215, SSU1355, SSU1773, SSU1915) through functional genomic screening, which induced robust immune responses, including IgG antibodies and cell-mediated immunity, and provided significant protection against virulent *S. suis*. The efficacy of these vaccines was highly dependent on the choice of adjuvants, with Carbopol-AddaVax and Emulsigen-D showing particular promise (55).

Innovative delivery methods have also shown potential. For instance, intranasal administration of the V5 multi-subunit vaccine, comprising SrtA, SspA, SLY (56), MRP, and ScpCL virulence factors with CpG ODN adjuvant, promoted robust mucosal immunity in mice, leading to significant clearance of *S. suis* from the nasopharynx and high homologous protection against systemic SS2 challenge (57). Despite these successes, some candidates like DPPIV and enolase (SsEno) failed to provide protection in mice, highlighting the need for further optimization (45). Proteomic and bioinformatic analyses identified 131 predicted surface proteins in *S. suis* human isolates, providing a resource for vaccine candidate testing, though *in vivo* immunogenicity and protective capacity were not evaluated (58).

Engineering approaches have shown potential in enhancing vaccine efficacy. BP formulations such as 38-BP-Enol and SSU1-BP-SSU2 induced significant antigen-specific humoral immune responses, with IgG titers reaching up to 1.0×10^7^, and provided 100% survival in vaccinated mice compared to 70% in control groups (59). Similarly, proteins like IF-2 and 1022 provided cross-protection against lethal doses of SS2 and SS9, with high antibody titers and significant protection in challenge experiments (60). Yet, a multicomponent vaccine composed of six conserved immunogens failed to demonstrate significant protection in piglets challenged with *S. suis* cps14 (61). In contrast, a trivalent protein vaccine (JointS) combined with a TLR4 agonist (MPLA) provided complete protection against SS2 infection in mice and good protection in piglets (62). Additionally, an engineered *E. coli* strain secreting *S. suis* antigens using the Tat pathway showed higher survival rates and milder clinical symptoms in vaccinated mice (63). Yet, the risk of gene transfer and environmental contamination must be carefully assessed. Multi-epitope vaccines (e.g., MVSS) (64) and MEASs (65) have also shown strong immunogenicity and partial protection in mouse models, with some formulations demonstrating superior efficacy. These studies highlight the potential of multi-subunit vaccines but also underscore the need for further optimization to achieve broad-spectrum protection.

## 4 Animal models in SS vaccine

**Figure 3:**
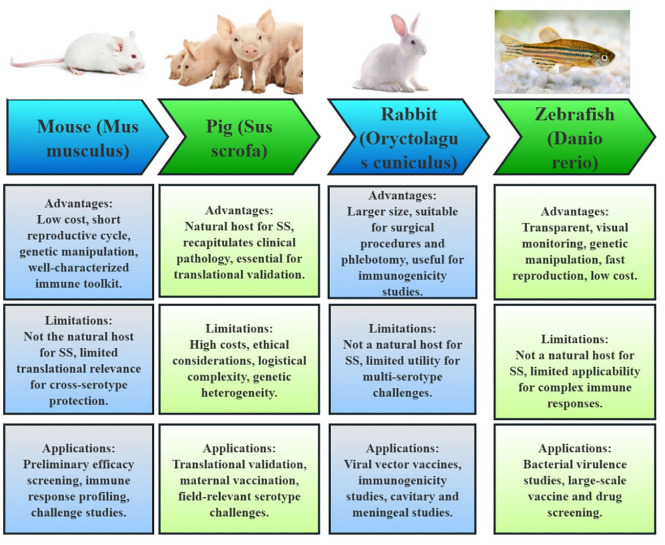
Animal models utilized in the development of *Streptococcus suis* subunit vaccines. The figure illustrates the key animal models employed to assess immunogenicity, protective efficacy, and safety of vaccine candidates. For each model, its advantages, limitations and applications are summarized, reflecting their distinct roles in vaccine evaluation.

In the development of subunit vaccines for *S. suis*, a variety of animal models have been employed to evaluate efficacy, immunogenicity, and protection. These models include mice, pigs, rabbits, and zebrafish, each with distinct roles, advantages, and limitations, as shown in **Figure 3**.

Mice are the most prevalent model used for preliminary efficacy screening, immune response profiling (antibody titers, cytokine levels), and challenge studies (66). The advantages of using mice include their low cost, short reproductive cycle, and ease of genetic manipulation. Additionally, well-characterized immune toolkits, such as the BALB/c and C57BL/6 strains, facilitate high-throughput screening of antigens and adjuvants. For instance, studies involving OMVs and *Salmonella* vectors have benefited from the scalability and genetic tractability of mice. Still, mice are not the natural host for SS, and their pathophysiology may not accurately mirror the disease in swine or humans. This limitation is particularly evident in conditions like SS meningitis, which is poorly modeled in mice. Furthermore, vaccines optimized in mice often fail to translate effectively to pigs, highlighting the limited translational relevance for cross-serotype protection.

Pigs are critical for translational validation, especially in evaluating maternal vaccination and field-relevant serotype challenges. As the natural host for SS, pigs recapitulate clinical pathology, including sepsis and meningitis, making them essential for assessing passive immunity (e.g., sow-to-piglet antibody transfer) and the efficacy of field strains (67). On the other hand, the use of pigs is constrained by high costs, ethical considerations, and logistical complexity. Additionally, the genetic heterogeneity of pigs may introduce variability in vaccine responses, complicating the interpretation of results.

Rabbits have niche applications (68), particularly in the study of viral vector vaccines like BoHV-4. Their larger size facilitates surgical procedures and phlebotomy, making them useful for immunogenicity studies of human-zoonotic strains. Despite these advantages, rabbits are not natural hosts for SS, and their immune mechanisms may not reflect those of swine. Moreover, rabbits have limited utility for multi-serotype challenges, as there are no established rabbit models for certain serotypes like SS7 and SS9.

Zebrafish represent an emerging model for rapid *in vivo* screening, exemplified by studies involving the joints trivalent vaccine combined with a TLR4 agonist. The transparency of zebrafish enables real-time imaging of immune responses, and their high fecundity and genetic tractability make them suitable for large-scale screening (69). Conversely, their evolutionary distance from mammals and the lack of adaptive immunity complexity limit their applicability for SS pathology and cross-protection studies.

## 5 Adjuvants for SS vaccine

Adjuvants play a crucial role in enhancing the immunogenicity and efficacy of bacterial vaccines (70). Over the years, various adjuvants have been explored in the development of subunit vaccines of SS2, with some showing significant enhancement of immune responses, while others have demonstrated limited or no effect, as displayed in **Table 1**. Freund’s Complete and Incomplete Adjuvants have been widely used in experimental studies and have shown strong enhancement of immune responses. For example, in studies involving the Sao protein and CPS-TT conjugates (34), Freund’s adjuvants induced high levels of IgG antibodies and robust Th1/Th2 immune responses, leading to significant protection against lethal challenges in mice and pigs. Quil-A, a saponin-based adjuvant, has been effective in enhancing mucosal and systemic immune responses (45). In studies using recombinant SaoA delivered via a *Salmonella* vector, Quil-A significantly boosted IgG and IgA levels, providing strong protection against SS2 and SS7. TiterMax Gold has shown significant enhancement of opsonizing IgG responses, particularly in CPS-based vaccines. For instance, CPS3 adjuvanted with TiterMax Gold induced strong opsonic IgG responses in mice, highlighting its potential for enhancing vaccine efficacy (21). Stimune has also been used in CPS-TT conjugate vaccines, inducing high levels of IgG antibodies and providing significant protection against lethal challenges in pigs. ISA 206 VG has been effective in enhancing IgG responses in multi-epitope vaccines, providing significant protection against SS2 and SS9 in mice (48).

On the other hand, some adjuvants have shown limited or no effect. Aluminum Hydroxide (AlOH), while commonly used, has shown limited efficacy in enhancing immune responses in some studies. For example, in CPS-based vaccines, AlOH failed to induce significant antibody responses, highlighting its potential limitations (49). Montanide ISA206, in some studies involving enolase and other subunit vaccines, did not provide significant protection, despite inducing high IgG titers. Polygen™ has also shown limited enhancement of immune responses in some studies (45), particularly in subunit vaccines like rDPPIV, where it failed to provide significant protection against SS2 challenge. Future research should focus on optimizing adjuvant formulations and exploring novel adjuvants to improve vaccine efficacy and safety.

## 6 Conclusions

Over the past decade, as depicted in **Figure 2** and **Table 2**, the evaluation of subunit vaccines for SS has identified several antigens that provide complete (100%) protection against lethal SS2 challenge in mouse and piglet models. Notably, only three antigens, SaoA, Ide r10, and Ide r10049, have demonstrated 100% protection in piglets, while other promising candidates like pS-Lpp-SaoA (delivered via OMVs), IgA1 protease, rIde-14009-1, Enolase, 6-GPD, 38-BP-Enol, and various combinations (e.g., MRP/GAPDH/DLD or SLY/Enolase/Sbp) have shown 100% protection in mice. However, most of these candidates have yet to achieve 100% protection in pigs. To bridge the efficacy gap between murine models and natural hosts, future research needs to focus intently on validation in pigs using physiologically relevant challenge models and route of immunization. Identifying immune correlates of protection, such as opsonic antibodies or mucosal IgA responses, in swine will be essential for guiding rational vaccine design and adjuvant selection. Future efforts should focus on identifying new immunogenic proteins and adjuvants to develop vaccines that can provide broader protection in target species such as swine.

**Table 2:** Summary of antigens conferring 100% protection against SS2 and evaluation of immune protection in the full text.

Antigen	Dose	No. of immunizations	Route	Adjuvant	Antibody response	Animal model	Challenge Dose/ Route	Protection Rate (SS2)	Reference
pS-Lpp-SaoA (OMVs)	10 µg	2x	Intraperitoneal (i.p.)	None	Highest IgG,IgG1,IgG2a; Balanced Th1/Th2; Strong Th17	BALB/c mice	8×LD_50_, i.p.	100%	(27)
SaoA	1x 10^9^ CFU	2x	Oral	Live vector (rSC0016)	Strong serum IgG, mucosal IgA, High IFN-γ, IL-4, IL-17A	BALB/c mice,Piglets	3x10^8^, i.p.	100%	(28)
SaoA	1 × 10^9^ CFU	2x	Oral	Attenuated S. *Choleraesuis* vector	High IgG/IgA ;Th1-biased (IgG2a > IgG1) -Induced cross-reactive OPA against SS2, SS7, SS9, SS1/2	BALB/c mice	10 × LD_50_ of SS2, SS7, SS9, SS1/2, i.p.	SS2: 100% SS7: 70% SS9: 60% SS1/2:60%	(32)
IgA1 protease	30 μg	2x	i.p.	Marcol 52	High IgG titers (IgG1/IgG2a)	BALB/c mice	2 × 10^9^, i.p.	100%	(32)
rIde-10 (Group A)	120 μg	2x	Intramuscular	X-Solve	IgG	Piglets	1×10^9^, intratracheal	100%	(39)
rIde-14009-1 (Group A)	50 μg	2x	Intramuscular	X-Solve	High IgG titers	Piglets	4×10^9^, intratracheal	100%	(39)
rS-ABC (full-length)	50 μg	2x	Subcutaneous injection	Freund’s adjuvant/ Incomplet adjuvant	Strong IgG response; IFN-γ/ IL-4	BALB/c mice	2-6×10^8^, i.p.	87.5%(SS2), 100% (SS9), 50% (SS7)	(42)
Enolase (via *S.choleraesuis*)	1×10^9^ CFU	2x	Oral	None	IgG/IgA, (Th1-dominant)	BALB/c mice	10×LD_50_ (SS2: 1.2×10^8^; SS7/SS9/SS1/2: varied), i.p.	100% (SS2), 80% (SS7), 100% (SS9)	(46)
6-GPD	1 ± 0.3 × 10^9^ CFU	2x	Oral	None	IgG, IgA	BALB/c mice	1.3 × 10^8^ or 2.6 × 10^8^ CFU, i.p.	100%	(47)
38-BP-Enol	6 μg	2x	Subcutaneous	Quil-A^®^	Ig>10^7^, IgG: >10^5^	C57BL/6 mice	8×10^7^ CFU, i.p.	100%	(59)
MRP, GAPDH,DLD	50 µg	3x	Subcutaneous	MPLA	High IgG titers	BALB/c mice	5×10^8^ CFU, i.p.	100%	(62)
SLY, Enolase,Sbp	10^18^ CFU	2x	Oral gavage	None	Not measured	Kunming Mice	1.5×10^9^, i.p.	100%	(63)
